# X-Ray Structure and *In Vitro* Anti-Tumoural Activity of the Dimeric Bis[(2-Phenyl-1,2-Dicarba-*Closo*-Dodecaborane-1-Carboxylato)-Di-n-Butyltin] Oxide

**DOI:** 10.1155/MBD.1997.75

**Published:** 1997

**Authors:** Edward R. T. Tiekink, Marcel Gielen, Abdeslam Bouhdid, Rudolph Willem, Vladimir I. Bregadze, Lidia V. Ermanson, Sergey A. Glazun

**Affiliations:** 1 Department of Chemistry The University of Adelaide South Australia 5005 Australia; 2 Department of General and Organic Chemistry Faculty of Applied Sciences Free University of Brussels (V.U.B.) Pleinlaan 2 Brussels B-1050 Belgium; 3 High Resolution NMR Centre Free University of Brussels (V.U.B.) Pleinlaan 2 Brussels B-1050 Belgium; 4 A. N. Nesmeyanov Institute of Organoelement Compounds Russian Academy of Sciences Vavilova Str. 28, V-334 Moscow 117813 Russia

## Abstract

X-ray diffraction studies reveal the structure of {[(2-C_6_H_5_-1,2-C_2_B_10_H_10_-1-COO)Bu_2_Sn]_2_O}_2_, **1**, to conform to the common motif found for {[(R′COO)R_2_Sn]_2_O}_2_ compounds. The dimer features a central Bu_2_Sn_2_O_2_ unit (two-fold symmetry) with the two Bu_2_Sn groups being linked via bridging oxygen atoms, each of which also carries an exocyclic Bu_2_Sn moiety. The two pairs of exo- and endo-cyclic tin atoms are each linked via an almost symmetrically bridging carboxylate ligand and the two remaining ligands coordinate an exocyclic tin atom only, in the monodentate mode. The *in vitro* anti-tumour activity of **1**, determined against a variety of cell lines, is compared with those of the corresponding 2-methylcarboranylacetate, derivative **2**, and with clinically used compounds.


### X-RAY STRUCTURE AND *IN VITRO* ANTI-TUMOURAL ACTIVITY OF THE DIMERIC BIS[(2-PHENYL-1,2-DICARBA-*CLOSO*-DODECABORANE-1-CARBOXYLATO)-DI-n-BUTYLTIN] OXIDE

Edward R. T. Tiekink[1], Marcel Gielen*[2], Abdeslam Bouhdid[2], Rudolph Willem[2,3], Vladimir I. Bregadze[4], Lidia V. Ermanson[4] , and Sergey A. Glazun[4]

[1] Department of Chemistry, The University of Adelaide, South Australia 5005, Australia
[2] Department of General and Organic Chemistry, Faculty of Applied Sciences
[3] High Resolution NMR Centre,
Free University of Brussels (V.U.B.), Pleinlaan 2, B-1050 Brussels, Belgium
[4] A. N. Nesmeyanov Institute of Organoelement Compounds, Russian Academy of Sciences, Vavilova Str. 28, V-334 Moscow, 117813 Russia

**Abstract**
X-ray diffraction studies reveal the structure of $\{[(2\text{-}C_6H_5\text{-}1,2\text{-}C_2B_{10}H_{10}\text{-}1\text{-}COO)Bu_2Sn]_2O\}_2$, **1**, to conform to the common motif found for $\{[(R'COO)R_2Sn]_2O\}_2$ compounds. The dimer features a central $Bu_4Sn_2O_2$ unit (two-fold symmetry) with the two $Bu_2Sn$ groups being linked via bridging oxygen atoms, each of which also carries an exocyclic $Bu_2Sn$ moiety. The two pairs of exo- and endo-cyclic tin atoms are each linked via an almost symmetrically bridging carboxylate ligand and the two remaining ligands coordinate an exocyclic tin atom only, in the monodentate mode. The *in vitro* anti-tumour activity of **1**, determined against a variety of cell lines, is compared with those of the corresponding 2-methylcarboranylacetate, derivative **2**, and with clinically used compounds.

### 1. Introduction

The *in vitro* anti-tumour activity of many compounds of the type $\{[(R'COO)Bu_2Sn]_2O\}_2$ has been determined [1-4]. Generally, the compounds are quite active. The synthesis and X-ray crystal structure of the dimeric bis-[(1,7-dicarba-*closo*-dodecaborane-1-carboxylato)-di-n-butyltin] oxide, $\{[(1,7\text{-}C_2B_{10}H_{11}\text{-}1\text{-}COO)Bu_2Sn]_2O\}_2$, has already been reported [5]. Its *in vitro* anti-tumour activity is less than those of several compounds of the type $\{[(R'COO)Bu_2Sn]_2O\}_2$, comparable to those of methotrexate and doxorubicin, but greater than those of 5-fluorouracil, cis-platin and carboplatin [5]. As a continuation of studies on organotin carboxylates, the synthesis, spectral characterisation, and *in vitro* anti-tumour activity of a bis-[(2-phenyl-1,2-carborane-1-carboxylato)di-n-butyltin] oxide, $\{[(2\text{-}C_6H_5\text{-}1,2\text{-}C_2B_{10}H_{10}\text{-}1\text{-}COO)Bu_2Sn]_2O\}_2$ (**1**), and of bis[1-(2-methyl-1,2-carboranyl)-1-acetato-di-n-butyltin] oxide, $\{[(2\text{-}CH_3\text{-}1,2\text{-}C_2B_{10}H_{10}\text{-}1\text{-}CH_2\text{-}COO)Bu_2Sn]_2O\}_2$ (**2**), are reported together with the X-ray structure of **1**.

### 2. Results and Discussion

### *2.1. Synthesis*

The novel dimeric bis[(2-phenyl-1,2-dicarba-*closo*-dodecaborane-1-carboxylato)-di-n-butyltin] oxide, compound **1**, was obtained from a 1:1 condensation of dibutyltin(IV) oxide with 2-phenyl-1,2-carborane-1-carboxylic acid. Its structure was determined by spectroscopic and X-ray diffraction methods. The 1-(2-methyl-1,2-carboranyl)acetate derivative, **2**, was synthesised using an analogous procedure. The $^1H$, $^{13}C$ and $^{117}Sn$ NMR spectra of the compounds display the usual duplicate resonances arising from the two heterotopic pairs of $Bu_2Sn$ moieties of dimeric dicarboxylatotetraorganodistannoxanes, $\{[(R'COO)R_2Sn]_2O\}_2$ [1-5].

### *2.2. Crystal and molecular structure of* $\{[(2\text{-}C_6H_5\text{-}1,2\text{-}C_2B_{10}H_{10}\text{-}1\text{-}COO)Bu_2Sn]_2O\}_2$, **1**

The molecular structure of **1** is illustrated in Figure 1 and selected interatomic parameters are collected in Table 1. The analysis shows that the structure is similar to those of a majority [6] of analogous $\{[(R'COO)R_2Sn]_2O\}_2$ compounds that usually feature a centrosymmetric $Sn_2O_2$ core (containing the endocyclic tin atoms) which associate, via the oxygen atom, to two exocyclic tin atoms leading to a partial ladder arrangement. The $\{[(R'COO)R_2Sn]_2O\}_2$ motifs differ in the mode of association of the four carboxylate ligands to the $Sn_4O_2$ moiety [6].

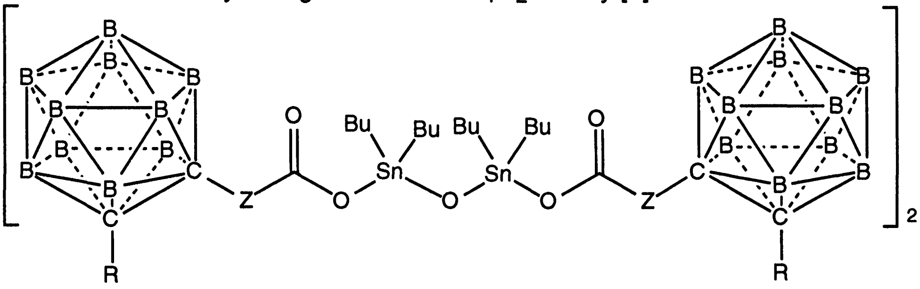

Compound **1** Z= - R = Ph
Compound **2** Z = $CH_2$ R = Me

The predominant motif shows two of the carboxylates to be bidentate, bridging a pair of endo- and exocyclic tin atoms, and each of the other two carboxylates to coordinate an exocyclic tin atom exclusively in the monodentate mode.

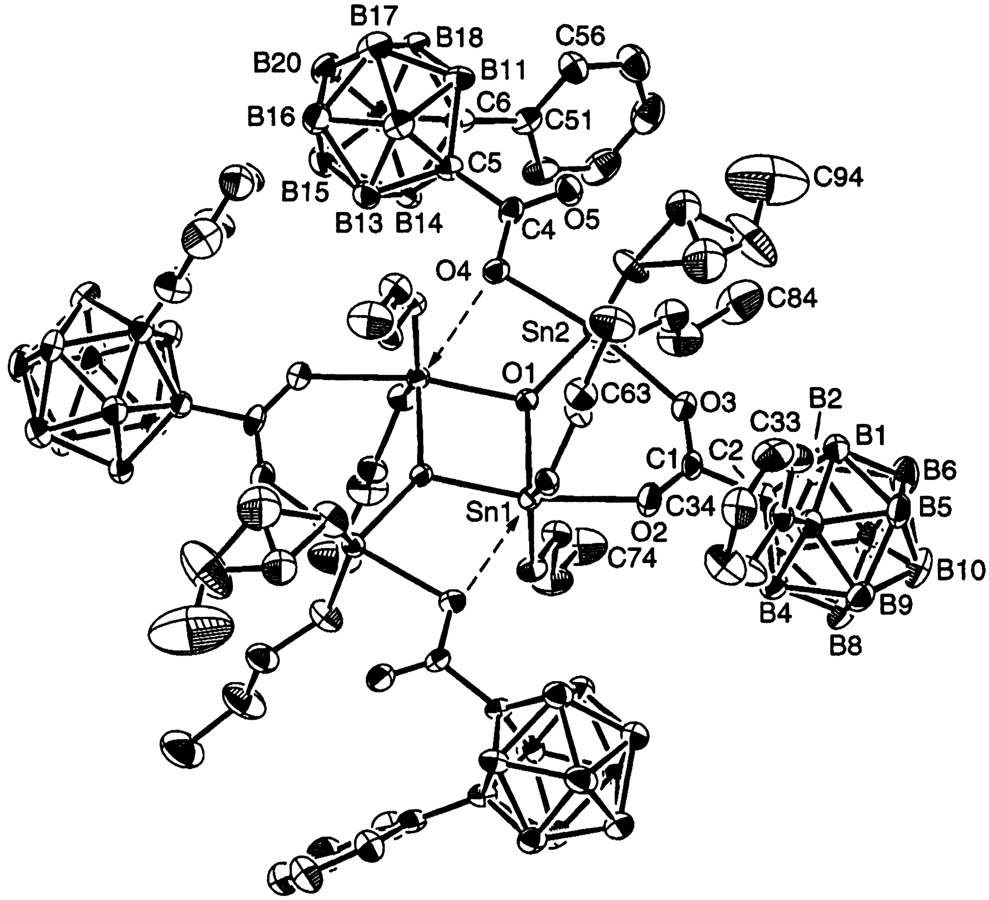

Figure 1: Molecular structure and crystallographic numbering scheme for $\{[(2\text{-}C_6H_5\text{-}1,2\text{-}C_2B_{10}H_{10}\text{-}1\text{-}COO)Bu_2Sn]_2O\}_2$. Hydrogen atoms are omitted for clarity. For the carboxylate moieties with C(1), the B(3) atom is obscured under C(31) and B(7) occupies a position in the B(5)-B(9) pentagonal plane. For the second carboxylate, B(12) lies above B(19) in the middle of the carborane as viewed.

This mode of coordination results in two five-coordinate, trigonal bipyramidal tin atom geometries. The structure of **1** is different from most of the other structures in that there is crystallographic two-fold symmetry, rather than a centre of inversion. It is noted that the core, mode of association of the carboxylate ligands and coordination geometries are as found for the common motif.

The structure of **1** is essentially molecular with no non-hydrogen contacts less than 3.6 Å. Within the molecule, one carboxylate ligand, i.e. containing C(1), bridges the Sn(1) and Sn(2) atoms forming almost symmetric Sn-O distances of 2.314(4) Å and 2.276(4) Å, respectively, and the second independent carboxylate anion forms a monodentate contact with Sn(2) such that Sn-O(4) is 2.244(4) Å. The Sn(1) atom lies 0.1335(4) Å out of the trigonal plane defined by the O(1), C(61) and C(71) atoms in the direction of the O(1)' atom, and Sn(2) lies 0.0315(4) Å out of the O(1), C(81) and C(91) plane towards the O(4) atom. The O(2)-Sn(1)-O(1)' and O(3)-Sn(2)-O(4) axial angles of 167.8(1)° and 169.7(2)°, respectively represent only small deviations from the ideal. Deviations from the ideal angles in the trigonal plane may be traced to close Sn...O intramolecular contacts. Thus, the O(4)' atom is separated by 2.734(4) Å from the Sn(1) centre and the Sn(2)...O(5) contact is 2.916(4) Å. These interactions are too long to represent significant bonding contacts; however, they are at least partly responsible for the widening of the C-Sn-C angles to 147.3(2)° and 141.6(3)°, respectively. If the Sn(1)-O(4)' interactions were considered significant, the molecular geometry may be described as being based on a $Sn_4O_4$ ladder, however, the relatively weak nature of these interactions suggests that a better description would be one based on a partially completed ladder.

TABLE 1. Selected interatomic parameters (Å, deg.) for **1**. Primed atoms are related by a crystallographic two-fold axis

| | | | |
|---|---|---|---|
| Sn(1)-O(1) | 2.062(4) | Sn(1)-O(1)' | 2.160(4) |
| Sn(1)-O(2) | 2.314(4) | Sn(1)-C(61) | 2.127(7) |
| Sn(1)-C(71) | 2.128(6) | Sn(2)-O(1) | 2.029(3) |
| Sn(2)-O(3) | 2.276(4) | Sn(2)-O(4) | 2.244(4) |
| Sn(2)-C(81) | 2.115(7) | Sn(2)-C(91) | 2.144(7) |
| O(2)-C(1) | 1.222(7) | O(3)-C(1) | 1.245(8) |
| O(4)-C(4) | 1.298(7) | O(5)-C(4) | 1.204(8) |
| C(1)-C(2) | 1.525(8) | C(4)-C(5) | 1.534(9) |
| O(1)-Sn(1)-O(1)' | 77.2(1) | O(1)-Sn(1)-O(2) | 90.6(1) |
| O(1)-Sn(1)-C(61) | 106.3(2) | O(1)-Sn(1)-C(71) | 105.1(2) |
| O(1)'-Sn(1)-O(2) | 167.8(1) | O(1)'-Sn(1)-C(61) | 98.4(2) |
| O(1)-Sn(1)-C(71) | 97.5(2) | O(2)-Sn(1)-C(61) | 84.7(2) |
| O(2)-Sn(1)-C(71) | 85.7(2) | C(61)-Sn(1)-C(71) | 147.3(2) |
| O(1)-Sn(2)-O(3) | 90.6(1) | O(1)-Sn(2)-O(4) | 80.1(1) |
| O(1)-Sn(2)-C(81) | 109.0(2) | O(1)-Sn(2)-C(91) | 109.3(2) |
| O(3)-Sn(2)-O(4) | 169.7(2) | O(3)-Sn(2)-C(81) | 86.5(2) |
| O(3)-Sn(2)-C(91) | 90.8(2) | O(4)-Sn(2)-C(81) | 92.4(2) |
| O(4)-Sn(2)-C(91) | 96.3(2) | C(81)-Sn(2)-C(91) | 141.6(3) |
| Sn(1)-O(1)-Sn(1)' | 102.8(1) | Sn(1)-O(1)-Sn(2) | 137.5(2) |
| Sn(1)-O(1)'-Sn(2)' | 119.7(2) | Sn(1)-O(2)-C(1) | 134.7(4) |
| Sn(2)-O(3)-C(1) | 137.4(4) | Sn(2)-O(4)-C(4) | 107.9(4) |
| O(2)-C(1)-O(3) | 127.3(6) | O(2)-C(1)-C(2) | 118.0(6) |
| O(3)-C(1)-C(2) | 114.7(5) | O(4)-C(4)-O(5) | 125.0(6) |
| O(4)-C(4)-C(5) | 114.7(5) | O(5)-C(4)-C(5) | 120.3(5) |

A partial crystal structure of **2** was obtained (see Experimental). The refinement halted at *R ca* 13 % and hence, the derived parameters are not reliable. The gross structural features were determined unambiguously, however, and showed that the common (i.e. centrosymmetric) motif found for $\{[(R'COO)R_2Sn]_2O\}_2$ is adopted by **2** [6].

### 2.3. In vitro anti-tumour activities

The compounds $\{[(2\text{-}C_6H_5\text{-}1,2\text{-}C_2B_{10}H_{10}\text{-}1\text{-}COO)Bu_2Sn]_2O\}_2$, **1**, and $\{[(2\text{-}CH_3\text{-}1,2\text{-}C_2B_{10}H_{10}\text{-}1\text{-}CH_2\text{-}COO)Bu_2Sn]_2O\}_2$, **2**, were screened *in vitro* against seven tumoural cell lines of human origin. The $ID_{50}$ values obtained, in ng/mL, are summarised in Table 2.

Against these cell lines, compounds **1** and **2** are significantly more active than 5-fluorouracil, cis-platin and carboplatin but less active than methotrexate and doxorubicin, whereas the parent carboxylic acid of **1** is inactive. The results for **1** and **2** are similar to those reported earlier for $\{[(1,7\text{-}C_2B_{10}H_{11}\text{-}1\text{-}COO)Bu_2Sn]_2O\}_2$ [5] and show that these carborane derivatives possess medium activity.

### 3. Experimental

### 3.1. Synthesis and purification

2-Phenyl-1,2-carborane-1-carboxylic acid, compound **3**, was prepared following Zakharkin et al. [7a] by the action of an equimolar amount of n-butyl lithium to 1-phenyl-1,2-carborane followed by the carboxylation of the obtained 2-phenyl-1-lithio-1,2-carborane with $CO_2$ and acid hydrolysis. (2-Methyl-1,2-carboran-1-yl)acetic acid, compound **4**, was also prepared following Zakharkin et al. [7b] by the action of equimolar amount of magnesium on 2-methyl-1-chloromethyl-1,2-carborane followed by carboxylation with $CO_2$ of the Grignard reagent obtained, followed by acid hydrolysis. Compound **1** was synthesised in benzene (150 mL) from di-n-butyltin oxide (664 mg) and **3** (500 mg). After 20 minutes of reflux, the clear solution obtained was refluxed for a further 5 h. The binary water/benzene azeotrope was distilled off with a Dean-Stark funnel. The benzenic solution obtained was distilled to 50% of its initial volume and the remaining solvent was evaporated *in vacuo*. The solid obtained, compound **1**, was purified by recrystallisation from methylene chloride/n-hexane. Yield: 76%, m.p.: 144-147 ∞C Compound **2** was synthesised similarly using **4**. Yield: 78%, m.p.: 185-188 ∞C.

TABLE 2. *In vitro* anti-tumour activities (ng/mL) of bis-[2-phenyl-1,2-dicarba-*closo*-dodecaborane-1-carboxylato)-tetra-n-butyltin] oxide, compound **1**, of the parent carboxylic acid, 1-phenyl-1,2-carborane-1-carboxylic acid, compound **3**, of bis-[1-(2-methyl-1,2-dicarba-*closo*-dodecaboranylacetato)-tetra-n-butyltin] oxide, compound **2**, and of some reference compounds used clinically, against MCF-7 and EVSA-T, two breast cancers, WiDr, a colon cancer, IGROV, an ovarian cancer, M19 MEL, a melanoma, A498, a renal cancer and H226, a non small cell lung cancer.

| Compounds | MCF-7 | EVSA-T | WiDr | IGROV | M19 MEL | A498 | H226 |
|---|---|---|---|---|---|---|---|
| **1** | 138 | 164 | 514 | 169 | 220 | 301 | 388 |
| **2** | 74 | 140 | 283 | 102 | 172 | 182 | 246 |
| **3** | 56500 | 1750 | 45100 | 42400 | 58300 | >60000 | 55000 |
| Carboplatin | 10500 | 4500 | 3500 | 2400 | 5500 | 1800 | 25000 |
| Cis-platin | 1400 | 920 | 1550 | 230 | 780 | 1200 | 3158 |
| 5-Fluorouracil | 350 | 720 | 440 | 850 | 310 | 340 | 5300 |
| Doxorubicin | 25 | 13 | 18 | 150 | 21 | 55 | 180 |
| Methotrexate | 15 | 26 | 7 | 20 | 18 | 16 | 70 |

### 3.2. Structure determination of $\{[(2\text{-}C_6H_5\text{-}1,2\text{-}C_2B_{10}H_{10}\text{-}1\text{-}COO)Bu_2Sn]_2O\}_2$, 1

Intensity data for a colourless crystal (0.10 x 0.26 x 0.39 mm) were measured at room temperature on a Rigaku AFC6R diffractometer fitted with MoKα radiation (graphite monochromator, $\lambda$ = 0.71073 Å) using the $\omega$:2$\theta$ scan technique so that $\theta_{max}$ was 27.5°. No decomposition of the crystal occurred during the data collection and the data set was corrected for Lorentz and polarization effects [8], and for absorption employing an empirical procedure (range of transmission factors: 0.942 - 1) [9]. A total of 12166 data (11932 unique) were collected and of these, 5729 that satisfied the $I \geq 3.0\sigma(I)$ criterion were used in the subsequent analysis.

Crystal data for **1**: $C_{68}H_{132}B_{40}O_{10}Sn_4$, $M$ = 2016.9, monoclinic, space group $C2/c$, $a$ = 28.877(6) Å, $b$ = 14.818(2) Å, $c$ = 23.275(2) Å, β = 92.26(1)°, $V$ = 9951(2) Å$^3$, $Z$ = 4, $D_{expt}$ = 1.346 g cm$^{-3}$, $F(000)$ = 4080, μ = 10.41 cm$^{-1}$.

The structure was solved by direct methods [10] and refined by a full-matrix least-squares procedure based on $F$ [8]. The non-hydrogen atoms were refined with anisotropic displacement parameters and hydrogen atoms were included in the model in their calculated positions (C-H 0.97 Å) with the exception for the C(91-94) butyl group. This latter group was found to have high thermal motion/disorder such that the C(92) atom was located over two positions of equal weight; hydrogen atoms were not included. The refinement was continued until convergence with sigma weights when $R$ = 0.038 and $R_w$ = 0.044. The maximum residual in the final difference map was 0.49 e Å$^{-3}$. The numbering scheme employed is shown in Fig. 1 which was drawn with ORTEP [11] at 35 % probability ellipsoids. Data manipulation were performed with the teXsan program [8] installed on an Iris Indigo work station. Other crystallographic details, comprising fractional atomic coordinates, thermal parameters, all bond distances and angles (in CIF format), and tables of observed and calculated structure factors are available on request (ERTT).

A partial structure of **2** was obtained. Data collection was as for **1**. Some butyl carbon atoms could not be located and the refinement was stopped at $R$ = 0.133, $R_w$ = 0.162.

Crystal data for **2**: $C_{52}H_{132}B_{40}O_{10}Sn_4$, $M$ = 1824.8, monoclinic, space group $P2_1/c$, $a$ = 16.36(1) Å, $b$ = 19.819(5) Å, $c$ = 14.69(2) Å, β = 106.86(8)°, $V$ = 4558(6) Å$^3$, $Z$ = 2, $D_{expt}$ = 1.329 g cm$^{-3}$, $F(000)$ = 1848, μ = 11.28 cm$^{-1}$.

### 3.3. NMR experiments

All NMR spectra were recorded in $CDCl_3$ solutions on a Bruker AC250 instrument, using a QNP probe tuned at 250.13, 62.93, and 89.128 MHz for $^1$H, $^{13}$C, and $^{117}$Sn nuclei, respectively. $^1$H and $^{13}$C resonances were referenced to the solvent peak at 7.24 and 77.0 ppm, while Ξ($^{117}$Sn) = 35.632295 [12] was used for the $^{117}$Sn resonances. Chemical shifts in ppm and coupling constants in Hz. Abbreviations: t = triplet; tq = triplet of quartet; m = complex pattern; nv = non visible.

### 3.4. Mössbauer spectra

Mössbauer spectra were obtained as described previously [13]. QS = quadrupole splitting; IS = isomer shift; $\Gamma_1$ and $\Gamma_2$ = line widths, all in mm/s.

### 3.5 . Characterization

Compound **1**:

Mössbauer: QS: 3.72, IS: 1.44, $\Gamma_1$: 0.99; $\Gamma_2$: 1.03; $^1$H NMR: α-, β- and γ-$CH_2$: 1.05-1.15, m; $CH_3$: 0.83 (t, 7) & 0.86 ppm (t, 7); $H_o$: 7.59-7.62, m; $H_{m+p}$: 7.24-7.46, m; $^{13}$C NMR: C-1 & C-2: 79.6 & 83.3; CO: 163.6; $C_i$: 131.7; $C_o$: 131.1; $C_m$: 128.4; $C_p$: 130.4; $C_\alpha$: 28.8 [$^1J(^{13}C-^{119/117}Sn)$: 677] & 30.4 [$^1J(^{13}C-^{119/117}Sn)$: 710]; $C_\beta$: 26.5 [$^2J(^{13}C-^{119/117}Sn)$: 40] & 26.8 [$^2J(^{13}C-^{119/117}Sn)$: nv]; $C_\gamma$: 26.8 [$^3J(^{13}C-^{119/117}Sn)$: 127] & 27.4 [$^3J(^{13}C-^{119/117}Sn)$: 132]; $CH_3$: 13.57 & 13.63 ppm; $^{117}$Sn NMR: -201.6 [$^2J(^{119}Sn-O-^{119/117}Sn)$: 131] & -189.3 ppm [$^2J(^{119}Sn-O-^{119/117}Sn)$: 125].

Compound **2**:

Mössbauer: QS: 3.53, IS: 1.36, $\Gamma_1$: 0.83; $\Gamma_2$: 0.83; $^1$H NMR: α-$CH_2$: 1.59-1.65, β- and γ-$CH_2$: 1.31-1.46, m; $CH_3$: 0.91 (t, 7) & 0.93 ppm (t, 7); C-$CH_2$: 3.06 (s); C-$CH_3$: 2.07 (s); $^{13}$C NMR: C-1 & C-2: 74.8 & 72.9; CO: 172.3; C-$CH_2$: 43.2; C-$CH_3$: 23.5; $C_\alpha$: 27.7 [$^1J(^{13}C-^{119/117}Sn)$: 670] & 30.9 [$^1J(^{13}C-^{119/117}Sn)$: nv]; $C_\beta$: 26.7 [$^2J(^{13}C-^{119/117}Sn)$: 42] & 26.8 [$^2J(^{13}C-^{119/117}Sn)$: 43]; $C_\gamma$: 26.7 [$^3J(^{13}C-^{119/117}Sn)$: 125] & 27.3 [$^3J(^{13}C-^{119/117}Sn)$: 120]; $CH_3$: 13.5 & 13.6 ppm; $^{117}$Sn NMR: -205.2 [$^2J(^{119}Sn-O-^{119/117}Sn)$: 122] & -201.0 ppm [$^2J(^{119}Sn-O-^{119/117}Sn)$: 116].

### 3.6. In vitro screening

The *in vitro* tests were performed as described previously [14].

**Acknowledgments**

We thank Dr. B. Mahieu for recording the Mössbauer spectra and Mrs. I. Verbruggen for recording the NMR spectra. We are grateful to Mr. H. J. Kolker, Dr. J. Verweij, Prof. Dr. G. Stoter, Dr. J. H. M. Schellens, Laboratory of Experimental Chemotherapy and Pharmacology, Department of Medical Oncology, Rotterdam Cancer Institute, NL - 3008 AE, Rotterdam, The Netherlands, and Dr. D. de Vos, Pharmachemie BV, NL-

2003 RN Haarlem, The Netherlands for the *in vitro* tests. This research was supported by INTAS (contract 93-659), by the Belgian "Fonds voor Kollektief Fundamenteel Onderzoek" (F.K.F.O. grant number 2.0094.94, R.W.), the Belgian "Nationale Loterij" (grant number 9.0006.93, R.W.), and the "Russian Foundation for Basic Research" (grant number 96-03-32883, V.B., L.E. & S.G.). The Australian Research Council is gratefully thanked for support of the X-ray facility (E.R.T.T.).

### References

1 M. Gielen, *Coord. Chem. Rev.*, **151** (1996), 41

2 M. Gielen, E. R. T. Tiekink, A. Bouhdid, D. de Vos, M. Biesemans, I. Verbruggen, and R. Willem, *Appl. Organomet. Chem.*, **9** (1995), 639

3 M. Gielen, A. Bouhdid, F. Kayser, M. Biesemans, D. de Vos, B. Mahieu, and R. Willem, *Appl. Organomet. Chem.*, **9** (1995), 251

4 M. Gielen, M. Bouâlam, B. Mahieu, and E. R. T. Tiekink, *Appl. Organomet. Chem.*, **8** (1994), 19.

5 M. Gielen, A. Bouhdid, R. Willem, V. I. Bregadze, L. V. Ermanson, and E. R. T. Tiekink, *J. Organomet. Chem.*, **501** (1995), 277.

6 *a*. E. R. T. Tiekink, *Appl. Organomet. Chem.*, **5** (1991), 1; *b*. E. R. T. Tiekink, *Trends in Organometallic Chemistry*, **1** (1994), 71.

7 *a* . L. I. Zakharkin, V. I. Stanko, A. I. Klimova, and Yu. A. Chapovsky, *Izv. Akad. Nauk SSSR, Ser. khim.*, 1963, 2236; *b* . L. I. Zakharkin and A. V. Grebennikov, *Izv. Akad. Nauk SSSR, Ser. khim.*, 1967, 696.

8 *teXsan, Single Crystal structure analysis software*, Version 1.6 (1993), Molecular Structure Corporation, The Woodlands, TX, USA.

9 N. Walker and D. Stuart, *Acta Crystallogr.*, **A39** (1983), 158.

10 P. T. Beurskens, G. Admiraal, G. Beurskens, W. P. Bosman, S. García-Granda, J. M. M. Smits, and C. Smykalla (1992), The DIRDIF program system, Technical Report of the Crystallography Laboratory, University of Nijmegen, The Netherlands.

11 C. K. Johnson, ORTEPII, Report 5136, (1976), Oak Ridge National Laboratory, TN, USA.

12 J. Mason, Multinuclear NMR, Plenum Press, New York, p. 627 (1987).

13 M. Bouâlam, R. Willem, M. Biesemans, B. Mahieu, J. Meunier-Piret, and M. Gielen, *Main Group Met. Chem.*, **14** (1991), 41.

14 M. Gielen, R. Willem, A. Bouhdid, D. de Vos, C. M. Kuiper, G. Veerman, and G. J. Peters, *In vivo*, **6** (1995), 59.

**Received: February 12, 1997 - Accepted: March 7, 1997 - Received in revised camera-ready format: March 7, 1997**

